# A dominance shift in arid savanna: An herbaceous legume outcompetes local C_4_ grasses

**DOI:** 10.1002/ece3.4188

**Published:** 2018-06-11

**Authors:** Thomas C. Wagner, Johanna Richter, David F. Joubert, Christina Fischer

**Affiliations:** ^1^ Department of Ecology and Ecosystem Management Restoration Ecology Technische Universität München Freising Germany; ^2^ Natural Resources and Spatial Sciences Namibia University of Science and Technology Windhoek Namibia

**Keywords:** *Crotalaria podocarpa*, dominance shift, encroachment, ontogenetic shift, plant–plant interaction, *Stipagrostis ciliata*

## Abstract

The characteristic vegetation structure of arid savannas with a dominant layer of perennial grass is maintained by the putative competitive superiority of the C_4_ grasses. When this competitive balance is disturbed by weakening the grasses or favoring the recruitment of other species, trees, shrubs, single grass, or forb species can increase and initiate sudden dominance shifts. Such shifts involving woody species, often termed “shrub encroachment”, or the mass spreading of so‐called increaser species have been extensively researched, but studies on similar processes without obvious preceding disturbance are rare. In Namibia, the native herbaceous legume *Crotalaria podocarpa* has recently encroached parts of the escarpment region, seriously affecting the productivity of local fodder grasses. Here, we studied the interaction between seedlings of the legume and the dominant local fodder grass (*Stipagrostis ciliata*). We used a pot experiment to test seedling survival and to investigate the growth of *Crotalaria* in competition with *Stipagrostis*. Additional field observations were conducted to quantify the interactive effect. We found germination and growth of the legume seedlings to be facilitated by inactive (dead or dormant) grass tussocks and unhindered by active ones. Seedling survival was three times higher in inactive tussocks and *Crotalaria* grew taller. In the field, high densities of the legume had a clear negative effect on productivity of the grass. The C_4_ grass was unable to limit the recruitment and spread of the legume, and *Crotalaria* did outcompete the putative more competitive grass. Hence, the legume is able to spread and establish itself in large numbers and initiate a dominance shift in savannas, similar to shrub encroachment.

## 1 INTRODUCTION

Savannas and savanna‐like ecosystems occupy a fifth of the earth’s land surface and almost 40% of southern Africa. As habitat for wildlife and rangelands, they are of high value for conservation, tourism, and pastoral production and ensure peoples livelihood in this region (Safriel et al., 2005). Apart from the common coexistence of grasses and trees, savannas cover a wide range of climatic conditions and hence they vary considerably. The majority of southern African savannas are semiarid or arid and receive less than 600 mm mean annual precipitation (MAP; Sankaran et al., 2005). Water is a limiting factor, and tree density is decreasing with lower MAP (D’Onofrio, Baudena, D’Andrea, Rietkerk, & Provenzale, 2015; Sankaran et al., 2005). Whereas at higher rainfalls trees are still abundant, the woody cover of the dryer savannas such as the thornbush savannas of Namibia is sparse. There the vegetation is determined by a dominant layer of tufted perennial C_4_ grasses in which trees and shrubs, mostly legumes, are sparsely interspersed. Annual grasses and forbs only occur during the rainy period in low abundances.

The characteristic vegetation structure in these water‐ and nitrogen‐limited environments is regulated and adjusted by competitive processes, mainly between grasses and trees (Bond, 2008; Cramer, Van Cauter, & Bond, 2010; Donzelli, De Michele, & Scholes, 2013; Ward, Wiegand, & Getzin, 2013) but also other species (Sasaki & Lauenroth, 2011). Thereby, it is generally assumed that the living grasses use their superior competitive abilities (Cech, Edwards, & Olde Venterink, 2010) to maintain their dominance by suppressing the germination, reducing the growth, and thus regulating the establishment of trees and other competing species (Bond, 2008; D’Odorico, Okin, & Bestelmeyer, 2012; Sankaran, Ratnam, & Hanan, 2004). However, some studies contradict these findings and found, for example, seedlings of *Acacia mellifera* to grow and establish even when growing within grass tussocks (Joubert, Smit, & Hoffman, 2012; Rothauge, 2011). Furthermore, under water‐ and nutrient scarcity, facilitative interactions between grass tussocks and legumes gain in importance (Brooker et al., 2008; Maestre, Callaway, Valladares, & Lortie, 2009), as the tussocks store humidity and provide nutrients in form of litter (Maestre, Bautista, Cortina, & Bellot, 2001). Consequently, dead or dormant grass tussocks have even been found to support the germination and growth of other species seedlings (de Dios, Weltzin, Sun, Huxman, & Williams, 2014; Synodinos, Tietjen, & Jeltsch, 2015). These facilitative and competitive processes are modulated by the intensity and distribution of rainfall and the availability of water within the growing period. Water availability does not only govern the germination of annuals and the rejuvenation of perennials but also influences the competitive balance between grasses and trees (Archer, Anderson, Predick, Schwinning, & Steidl, 2017; Joubert, Smit, & Hoffman, 2013; Kulmatiski & Beard, 2013; Woods, Archer, & Schwinning, 2014). If this well‐balanced equilibrium is disturbed, sudden dominance shift can occur. Seedling of native shrubs or trees can gain the upper hand and lead to shrub encroachment (Sankaran et al., 2004; Scholes & Archer, 1997; Walter, 1953) or an “increaser” forb or grass can prevail and start a massive spreading (Smet & Ward, 2005; Vesk & Westoby, 2002). Both types of dominance shift in savannas are often associated with disturbances such as overgrazing or frequent fires. Generally, such dominance shifts are induced by any process or factor that either weakens the grass’s competitive abilities (e.g., through overgrazing; Ward, 2005), increases germination and recruitment rates of the competing species (Synodinos et al., 2015; Van Auken, 2009), or strengthens their seedlings (e.g., higher rainfalls, elevated CO_2_; Bond & Midgley, 2000; Kulmatiski & Beard, 2013).

In particular, shrub encroachment has become more frequent throughout the last decades (Higgins & Scheiter, 2012; O’Connor, Puttick, & Hoffman, 2014). Among disturbances such as land management changes and altered fire or grazing regimes, this increase is also attributed to higher atmospheric CO_2_‐levels (du Toit & O’Connor, 2014), as elevated CO_2_ concentrations decrease the photosynthetic disadvantage of C_3_ plants or even favor them against the generally better performing C_4_ grasses in hot environments (Bond & Midgley, 2000). In nutrient‐limited savannas, increased CO_2_ particularly favors legumes, as they are able to fix atmospheric nitrogen, strengthening their competitive abilities and affecting the competitive balance between grasses and legumes (Ward, Hoffman, & Collocott, 2014).

Although most dominance shifts in savannas known today are disturbance induced and the majority involve woody species (Eldridge et al., 2011; Sankaran et al., 2004; Scholes & Archer, 1997), there are a growing number of reports from farming communities in southern Africa about recent increases of native forbs obviously unrelated to disturbances. Likewise, the native herbaceous legume *Crotalaria podocarpa* has encroached parts of Namibia’s escarpment region with considerable impact on fodder grass production and the lands carrying capacity (Wagner, Hane, Joubert, & Fischer, 2016). With our study, we investigate the competitive balance between this legume and the dominant local fodder grass *Stipagrostis ciliata* and try to establish the causes for this recent spread of *Crotalaria podocarpa*. We carried out a controlled pot experiment to characterize the interaction between the C_4_ grass *Stipagrostis* and the legume *Crotalaria* and supplemented this experiment with long‐term field observations. We thereby hypothesize that:

1. *Crotalaria* is facilitated by *Stipagrostis* tussocks;
2. once *Crotalaria* is established, it has a negative effect on *Stipagrostis;*
3. a negative effect on *Stipagrostis* is dependent on the *Crotalaria* density and independent of rainfall.

## 2 MATERIAL AND METHODS

### 2.1 Study area

The collection of soil and plant material for our interaction experiment and the field study took place on the farm Rooiklip, Khomas (S 23°24′23.29′′, E 016°03′37.35′′), situated 1,000 m a.s.l. in Namibia’s lower great escarpment. Soils are predominantly shallow, nutrient‐poor calcisols with a coarse texture and a high proportion of stones and sand (Wagner et al., 2016). The climate is hot‐arid (Mendelsohn, Jarvis, Robertson, & Roberts, 2009). The scarce rainfall occurs predominantly between October and April with a pronounced precipitation maximum in February and March that defines the main growing season. Mean annual precipitation is 120 mm, while the mean precipitation during the growing season is 80 mm (Wagner and Fischer, unpublished data). The last decade (2005‐2014) was characterized by above‐average rainfall and prolonged humid periods (Appendix, Figure S1), with a mean annual rainfall of 242 ± 57 mm and a mean February to March precipitation of 170 ± 42 mm. The vegetation is dominated by tufted perennial C_4_ grasses of the genus *Stipagrostis*, mainly *Stipagrostis ciliata* (nomenclature according to De Winter, 1962; hereafter referred to as *Stipagrostis*). Together with the related *S. uniplumis,* it constitutes the main source of forage in Namibia’s escarpment region (Juergens, Oldeland, Hachfeld, Erb, & Schultz, 2013; Müller, 2007). On the study site, *Stipagrostis* forms a light matrix of 1.5–2 tussocks/m^2^ (Wagner et al., 2016) and its cover is considerably varying with seasonal rainfall. Loosely interspersed are perennial shrubs and occasionally trees. With sufficient rainfall, these perennials are complemented by annual grasses and forbs, including *Crotalaria podocarpa*. The area has not been used for farming or livestock‐keeping for over two decades, but the area is used by free‐roaming grazing wildlife such as zebra or gemsbok.

### 2.2 Study species

*Crotalaria podocarpa* DC (hereafter referred to as *Crotalaria*) is an annual, herbaceous legume that is widespread in southern Africa (Polhill, 1968). It occurs on both sandy and stony soils, is well adapted to arid conditions, and has the capability to fix nitrogen (Jourand et al., 2005). Due to its content of pyrrolizidine alkaloids and flavonoids, *Crotalaria* is unpalatable to livestock (Wanjala & Majinda, 1999). The growth, number of flowers, and seed set of *Crotalaria* vary considerably with the amount of rainfall, and the plant produces a high number of seeds that are viable for more than 7 years. Its comparatively large and heavy seeds are primarily dispersed by explosive dehiscence and reach a distance of about 5 m around the mother plant. Secondary long‐distance dispersal is rare and probably related to scatter hoarding by ants and small mammals (Fischer, Kollmann, & Wagner, 2015). *Crotalaria* is part of the local plant community of Namibia’s escarpment region where it normally occurs in moderate numbers. Starting in 2008, in the course of several years of above‐average rainfall, a considerable proliferation of *Crotalaria* has been observed in Namibia’s escarpment region (Wagner et al., 2016).

### 2.3 Interaction experiment: competition and facilitation

To characterize the interaction between active and inactive (dead or dormant) *Stipagrostis* tussocks and *Crotalaria* seedlings, we carried out a greenhouse experiment under natural temperature regime and simulated natural rainfall conditions in March 2015. In this pot experiment, we tested the following treatments, each with 15 replicates: (1) *Crotalaria* seedlings, (2) *Crotalaria* seedlings within inactive *Stipagrostis* tussocks, (3) *Crotalaria* seedlings within active *Stipagrostis* tussocks, and (4) active *Stipagrostis* tussocks. Both inactive and active grass tussocks had approximately 5 cm basal diameter each and were clipped to 5 cm to ensure similar starting conditions and avoid shading. Three *Stipagrostis* tussocks each were planted in one pot (size 220 × 150 × 150 mm) to ensure intraspecific competition. Soil material and *Stipagrostis* tussocks were taken from an area of the farm that was unaffected by *Crotalaria* encroachment and free of *Crotalaria* seeds. *Crotalaria* seeds were collected in 2014 in *Crotalaria*‐affected areas. Seeds were mechanically scarified with a scalpel and left for 24 h in petri dishes with 1 mm water to soak. Afterward, 10 swollen seeds with emerged radicle were evenly spaced 1 cm into the soil or the organic base of the grass tussock. Immediately after sowing, all pots were watered with a watering can equivalent to a rainfall event of 10 mm (equates to 330 ml/pot) for 3 subsequent days, and again after 5, 10, and 20 days. As *Crotalaria* typically reaches its flowering stage between 20 and 40 days (Wagner, unpublished data), we measured the number of surviving *Crotalaria* seedlings, maximum height of the emerging plants, and the maximum length of *Stipagrostis* culms for 34 consecutive days. Seedlings, which did not yet have cotyledons, were wilting, or damaged were excluded from the measurement. Complementary to this, we characterized the water retention capacity of the respective substrates bare soil, soil with inactive grass tussocks, and soil with active tussocks using time‐domain reflectometry (Ledieu, De Ridder, De Clerck, & Dautrebande, 1986; Robinson, Jones, Wraith, Or, & Friedman, 2002). Each pot was measured at 7 hr in the morning with three repeats over 10 days after a simulated rain event of 10 mm.

### 2.4 Field observations: *Stipagrostis* productivity and density

We used complementary field data to characterize the interaction between *Crotalaria* and *Stipagrostis* tussocks. Our field observation was carried out on 20 long‐term observation plots of 10 × 10 m half of which were affected by *Crotalaria* encroachment (Wagner et al., 2016). All plots had identical soil types, similar soil texture, and initially the same vegetation composition and structure (Table S1) with a *Stipagrostis* tussock density of about 2 tussocks/m². All plots were within a 2 km range to ensure similar rainfall conditions. Sampling took place at the end of the growing season in April each year between 2009 and 2015. The number of individuals of *Crotalaria* and *Stipagrostis* was counted and the area covered per individual (m²) was calculated from plant diameter. *Stipagrostis* tussocks were partitioned into active tussocks (showing green culms) and inactive (dead or dormant) tussocks. *Crotalaria* individuals were included if at least one pinna was developed and differentiated into individuals growing within or outside *Stipagrostis* tussocks of the two activity states. Individual cover was used as proxy for seasonal biomass production (Carlyle, Fraser, & Turkington, 2014; Henschel, Burke, & Seely, 2005). To verify the facilitation of *Crotalaria* seedlings by *Stipagrostis* tussocks under field conditions, we compared the ratio of seedlings growing within tussocks to seedlings growing on open soil with the ratio of area occupied by tussocks to area taken up by open soil.

### 2.5 Data analysis

**Figure 1:**
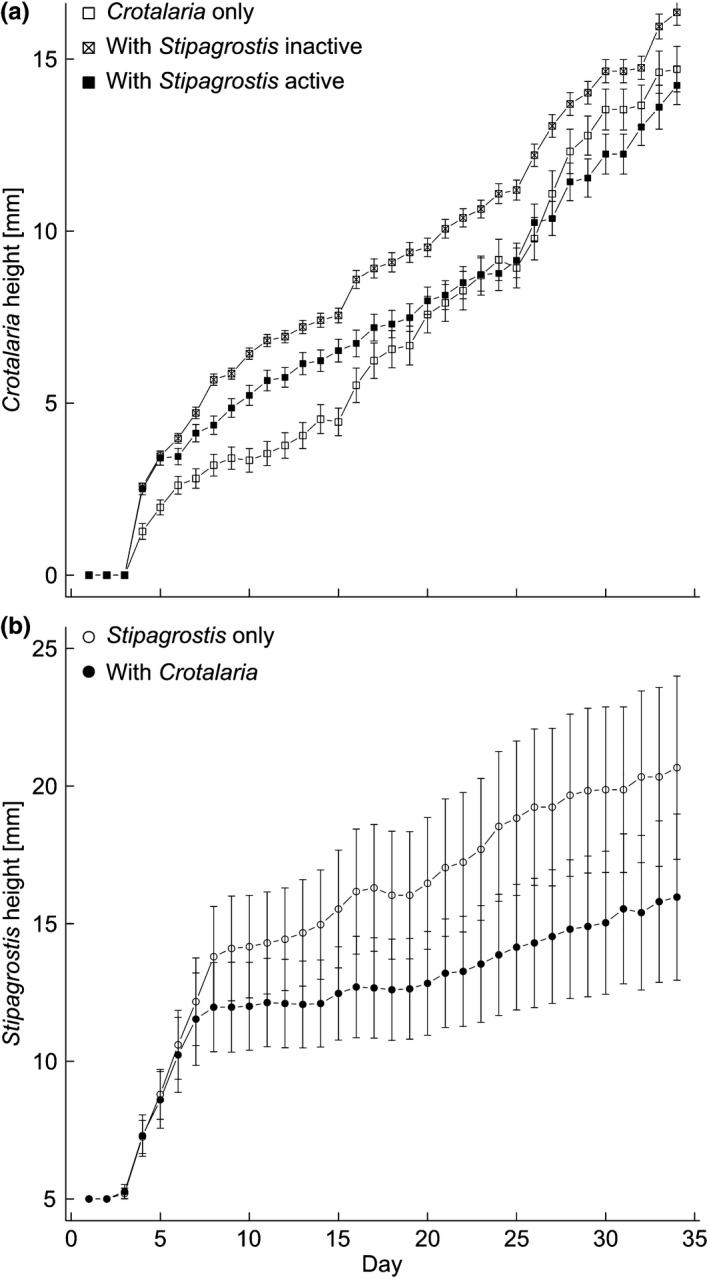
Interaction between *Crotalaria* and *Stipagrostis*. (a) Growth of *Crotalaria* growing alone, facilitated by inactive (dormant or dead) *Stipagrostis* tussocks and under competition with active *Stipagrostis*. (b) Growth of *Stipagrostis* alone and under competition with *Crotalaria*. Bars indicate standard error

Statistical analysis was performed with R 3.2.1 (R Core Team, 2015). Daily growth of *Stipagrostis* and *Crotalaria* during the interaction experiment was modeled using a linear mixed‐effects model (*lme*; library *nlme* version 3.1–120, Pinheiro, Bates, DebRoy, & Sarkar, 2015) with day and treatment as explanatory variables, including interactions. Pots were used as a random factor. *Stipagrostis* growth was modeled calculating two separate models for Days 3–7, and for Days 8–34, as growth rates of both treatments characteristically changed on Day 8 (see Figure 1). Comparison of seedling survival and final growth was made by permutational *t* tests (*perm.t.test*, library *RVAideMemoire* version 0.9–64; Hervé, 2015).

Data obtained from the field experiment were used to determine the direction and intensity of the interaction between *Crotalaria* and *Stipagrostis* and to elucidate the effect of *Crotalaria* density on it. Fisher’s exact test was used to compare the proportion of *Crotalaria* seedlings growing within or outside tussocks with the respective ratio of area taken up by tussocks and open soil. To quantify the interaction between *Crotalaria* and *Stipagrostis*, we calculated the Neighbor‐Effect Intensity Index (NInt_A_) introduced by Díaz‐Sierra, Verwijmeren, Rietkerk, de Dios, and Baudena (2017) based on the average tussock area of *Stipagrostis* for each year. NInt_A_ is defined as 2 *(*dB*)/(*B*_*0*_+|*dB*|), with *B*_*0*_ **= tussock area of *Stipagrostis* growing alone and *dB* = *B*_*0*_–tussock area of *Stipagrostis* growing with *Crotalaria* as competitor. Thus, a negative value of NInt_A_ indicates a competitive effect of *Crotalaria* on the *Stipagrostis,* whereas a positive value indicates facilitative effects. Possible correlation between NInt_A_ and annual rainfall was rejected using Spearman’s correlation coefficient (*r* = 0.31, *p* = 0.54; Figure S2). We further quantified the effect of *Crotalaria* density on the productivity of *Stipagrostis* tussocks in relation to rainfall (average tussock area/seasonal rainfall) using a linear mixed‐effects model with plot as a random factor to ensure independence of errors with respect to temporal autocorrelations (Pinheiro & Bates, 2000). Rainfall lower than 40 mm was excluded in this model, as here the area covered by grass tussocks was reduced to their basal area and due to a high frequency of outliers. To obtain normality of variances, response, and explanatory, variables were log‐transformed.

**Table 1:** Rainfall, *Stipagrostis* tussock area and Neighbor‐Effect Intensity Index NInt_A_ of *Crotalaria* on *Stipagrostis* productivity (tussock area) between 2009 and 2015 based on the average individual cover of tussocks on affected and unaffected field sites

Year	Rainfall [mm]	*Stipagrostis* tussock area on affected sites [m^2^]	*Stipagrostis* tussock area on unaffected sites [m^2^]	Ratio affected/unaffected [%]	NInt_A_
2009	273	0.177 ± 0.006	0.253 ± 0.019	70.0	−0.462
2011	405	0.182 ± 0.006	0.285 ± 0.015	63.9	−0.535
2012	92	0.099 ± 0.007	0.173 ± 0.006	57.2	−0.599
2013	20	0.010 ± 0.002	0.018 ± 0.003	55.6	−0.615
2014	162	0.034 ± 0.009	0.115 ± 0.010	29.6	−0.827
2015	39	0.027 ± 0.004	0.039 ± 0.006	69.3	−0.471

## 3 RESULTS

### 3.1 Results of the interaction experiment

In our interaction experiment, seedling survival and growth of *Crotalaria* were clearly facilitated by inactive *Stipagrostis* tussocks. Grass tussocks were able to maintain soil humidity longer than bare soil. The moisture content of bare soil dropped below the permanent wilting point of coarse sand (~2%) three days after the rain event. Active tussocks maintained a moisture level above the permanent wilting point for 5 days and inactive tussocks even for 6 days (Figure S3). All grass tussocks maintained their initial activity state during the whole experiment.

Time and maximum number of germinated *Crotalaria* seeds that did reach a measurable seedling stage (at least cotyledons developed, size >20 mm) varied considerably between the treatments. From 150 seeds planted on bare soil, only 40 reached this stage after 10 days. Seeds planted in *Stipagrostis* tussocks reached this stage 2 days earlier and survived in higher numbers, with 70 seedlings in active tussocks and even 109 seedlings in inactive tussocks (Table S2). Overall seedling survival was three times higher when growing in inactive tussocks (mean ± *SE*: 62.7 ± 8.1%) than when growing alone (18.7 ± 6.0%) or in active *Stipagrostis* tussocks (19.3 ± 7.6%). After 34 days, *Crotalaria* growing in inactive *Stipagrostis* tussocks was taller (16.2 ± 0.4 cm) than when growing alone (14.1 ± 0.7 cm; *t *=* *−2.57, *p* < 0.05) or together with active *Stipagrostis* (14.3 ± 0.6 cm; *t *=* *1.74, *p* = 0.09). *Crotalaria* growth rates (Figure 1a) were similar for all treatments, with a constant rate of 0.46 ± 0.01 cm/day for *Crotalaria* growing alone, a slightly lower growth rate when growing in inactive grass tussocks (0.40 ± 0.01 cm/day), and a somewhat lower growth rate when growing in active tussocks (0.35 ± 0.01 cm/day; Table S3).

*Stipagrostis* showed no growth for the first 2 days. Between Day 3 and Day 7, *Stipagrostis* growing without *Crotalaria* and *Stipagrostis* under competition with *Crotalaria* both had the same high growth rate of 1.55 ± 0.26 cm/day, but from Day 8 on, growth rates slowed considerably and exhibited significant differences between *Stipagrostis* without competition with 0.28 ± 0.05 cm/day and *Stipagrostis* under *Crotalaria* competition with 0.16 ± 0.03 cm/day (Figure 1b, Table S4). At Day 34, *Stipagrostis* growing alone was with 20.67 ± 4.47 cm significantly (*t *=* *3.08; *p* < 0.01) taller than *Stipagrostis* growing together with *Crotalaria* which reached only 15.97 ± 4.07 cm.

### 3.2 Results of the field observations

During the whole study, only moderate grazing through zebra occurred, but no exceptional herbivory event, for example, through arthropods, was recorded. The number of *Crotalaria* seedlings growing within *Stipagrostis* tussocks was disproportionally high compared to the number of seedlings growing outside grass tussocks: On average, 60.0 ± 1.7% of all *Crotalaria* seedlings were found to grow within grass tussocks, which covered only 3% of the plot area. The Neighbor‐Effect Intensity Index NInt_A_ of *Crotalaria* on *Stipagrostis* was consistently negative over the whole study period, indicating a pronounced competitive effect of *Crotalaria* on *Stipagrostis* (Table 1).

**Figure 2:**
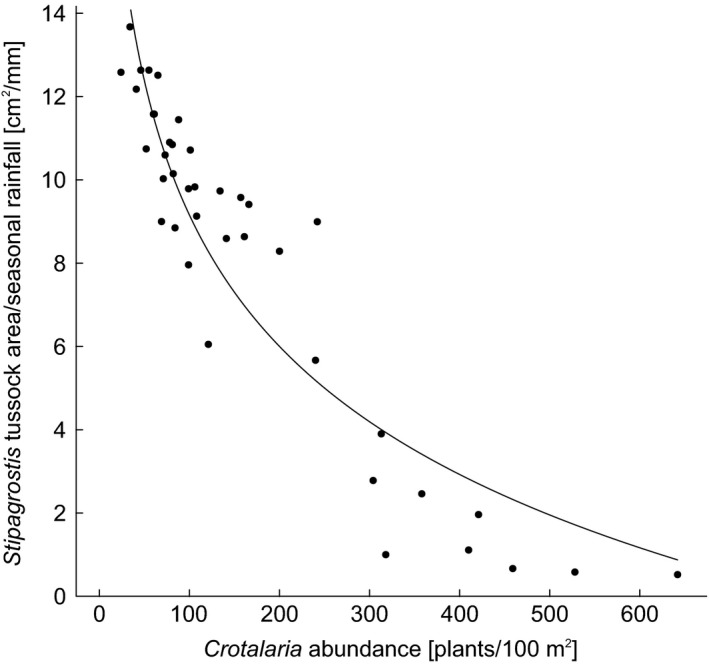
Effect of *Crotalaria* density on *Stipagrostis*: area/rainfall of active tussocks decreases with increasing *Crotalaria* abundance. The predicted line from the linear mixed‐effects models is shown

The area covered by active *Stipagrostis* tussock relative to seasonal rainfall exhibited a significant negative relationship with *Crotalaria* density (Estimate ± *SE*: −0.04 ± 0.00, *t*_29_ = −14.58, *p* < 0.001; Figure 2).

## 4 DISCUSSION

We found neutral or even facilitative effects of *Stipagrostis* on *Crotalaria*. Inactive (dead or dormant) grass tussocks clearly increased growth and survival of *Crotalaria* seedlings, whereas active grass tussocks did not affect seedling survival and growth of *Crotalaria*. Contrastingly, the effect of *Crotalaria* on *Stipagrostis* was consistently negative and increased with *Crotalaria* density, independent of rainfall.

Our interaction experiment covered the crucial early stages that are decisive for the successful recruitment of the legume (Bond, 2008; Harper, 1977; Wiegand, Saltz, & Ward, 2006). The interaction between *Stipagrostis* and *Crotalaria* was both facilitative and competitive, but clearly to the benefit of *Crotalaria* and to the detriment of the grass. During the growth of the legume, the interaction between *Crotalaria* and *Stipagrostis* undergoes a shift, and, while seedlings are facilitated by the grass, the legume later outcompetes or at least restricts (growth reduction) its former nurse plant. Similar ontogenetic shifts under arid conditions involving a grass as nurse plant have only been described for shrubs, such as *Stipa tenacissima* and *Lepidium subulatum* (Soliveres, Desoto, Maestre, & Olano, 2010) or *Agrostis magellanica* and *Azorella selago* (Roux, Shaw, & Chown, 2013). In our case, the effect of inactive *Stipagrostis* tussocks on *Crotalaria* seedlings was solely positive. Consistent with a study on the passive facilitation of saplings of *Prosopis velutina* by the C_4_ grass *Heteropogon contortus* (de Dios et al., 2014), seedling survival was clearly facilitated by inactive grass tussocks. During the early stages, *Crotalaria* seedlings growing in active or inactive grass tussocks developed more quickly and survived better than those on bare soil. This positive effect is probably due to the prolonged higher soil humidity that is maintained within the grass tussocks. After the eighth day, when the active *Stipagrostis* tussocks have finished their initial rapid growth phase, the number of *Crotalarias* growing in active tussocks is reduced and at the end of the experiment matches those of *Crotalarias* growing on bare soil.

The facilitation by grass tussocks was further corroborated by our field observations, where the density of *Crotalaria* seedlings in grass tussocks was up to 20 times higher than on bare soil. However, the growth of surviving *Crotalarias* was not affected. Even when growing in competition with active *Stipagrostis,* no negative effect on *Crotalaria* could be shown. Seedling survival and growth rate of *Crotalaria* growing within tussocks were not significantly different from those when growing alone (cf. Joubert, 2014 for *Acacia reficiens*). Nonetheless, at least at the early recruitment stages of *Crotalaria*, the active grass tussocks were able to exert some control on the recruitment of *Crotalaria* and thereby compensate for the facilitative effects. The impact of *Crotalaria* on *Stipagrostis* in contrast was consistently negative. Contrary to other studies on seedling competition (February, Higgins, Bond, & Swemmer, 2013), *Crotalaria* significantly reduced the growth rate of *Stipagrostis*. This negative effect of *Crotalaria* on *Stipagrostis* extends over the later growth stages of *Crotalaria,* increases with higher legume density, and is, other than the recruitment of *Crotalaria*, not compensated for by higher rainfalls and water availability. Thereby, our interaction experiments support the findings of Maestre, Bautista, and Cortina (2003) who found seedlings of legumes to grow largely unaffected in living grass tussocks under stressful conditions similar to those of our study region. In the case of *Crotalaria,* the C_4_ grass *Stipagrostis* is not, or probably no longer able to exert its supposed dominance (February et al., 2013; Riginos, 2009; Sankaran et al., 2004; Scholes & Archer, 1997) over the legume and suppress its recruitment. This clearly contradicts the widespread opinion that grasses are able to outcompete legumes and encroachers due to their competitive advantage in water‐limited environments (O’Connor et al., 2014; Riginos, 2009).

This raises the question why such a dominance shift involving *Crotalaria* has not been observed before. As classic disturbances such as increased grazing, herbivory events, or fire can be ruled out, we propose two possible explanations: Either the competitive capabilities of *Crotalaria* have recently gained in strength due to more favorable conditions or they are still unchanged but the incidence of competition has changed. In the latter case, the elevated rainfalls between 2007 and 2011 (Figure S1) might be a trigger. Higher rainfall is associated with higher seed production and generally better seedling survival of encroaching species (Kraaij & Ward, 2006; Oldeland, Dreber, & Wesuls, 2010; Roques, O’Connor, & Watkinson, 2001). Taking into account *Crotalarias* high seed production, its extended seed viability, and dispersal by explosive dehiscence (Fischer et al., 2015), above‐average rainfall will automatically be linked to a higher probability and number of seeds ending up in grass tussocks after dispersal. Our pot experiment has shown that the grass tussocks facilitate early establishment of *Crotalaria* and that the grass is unable to outcompete 3–4 *Crotalaria* seedlings per tussock. Our field data clearly demonstrate that the negative effect of *Crotalaria* on *Stipagrostis* increases with *Crotalaria* density. Consequently, there is now not only a higher probability of grass tussocks being faced with competing *Crotalaria,* but the affected tussocks may also have to compete with a higher number of *Crotalarias*, as more seedlings survive. This effect is further intensified as the perennial grass tussocks may already be weakened by competition during the preceding season.

Regarding a strengthening of *Crotalarias* competitive abilities, we may also speculate about a possible role of elevated atmospheric CO_2_ levels. Increased atmospheric CO_2_ has been recently identified to be an important determinant of encroachment processes (Higgins & Scheiter, 2012; Kulmatiski & Beard, 2013; O’Connor et al., 2014; Ward et al., 2014) as it favors C_3_ over C_4_ plants. Possible nutrient deficiencies can be compensated for by nitrogen fixation, provided temperatures are not too high, and there is sufficient water available (Sita et al., 2017). Once the seedlings have survived the early stages, they might then be able to grow more quickly and eventually overgrow the grasses. Already impaired by competing with a number of *Crotalaria* seedlings, the light‐sensitive grass tussocks (Zimmermann, Higgins, Grimm, Hoffmann, & Linstädter, 2010) later are additionally affected by shading and might be weakened even more, further contributing to a vicious circle at the end of which the dominance shift occurs.

## 5 CONCLUSION

The native herbaceous legume *Crotalaria podocarpa* does not only withstand the competition from the perennial C_4_ grass *Stipagrostis ciliata* but also has a pronounced negative effect on the grass. Likely due to the high number of seeds produced after higher rainfall, *Crotalaria* is able to prevail over the grass and initiate a dominance shift in the arid savanna of Namibia’s escarpment region. The ramifications of this shift resemble those of shrub encroachment or invasion, but it involves a native and herbaceous legume and is obviously not triggered by disturbance.

## CONFLICT OF INTEREST

None declared.

## AUTHOR CONTRIBUTIONS

TCW, CF, and DJ conceived and designed the experiments. JR and TCW performed the experiments and collected the data. CF and TCW analyzed the data. TCW and CF wrote the manuscript. All authors contributed critically to the draft and gave approval for publication.

## DATA ACCESSIBILITY

Data will be made available through OSF.
