# Supplementary material for: A dominance shift in arid savanna: An herbaceous legume outcompetes local C4 grasses
Source: Ecol Evol. 2018 Jun 11;8(13):6779–87. doi: 10.1002/ece3.4188 (PMC6053561; doi:10.1002/ece3.4188)
Supplement: Supplementary file 1 [file ECE3-8-6779-s001.docx]

**APPENDIX**


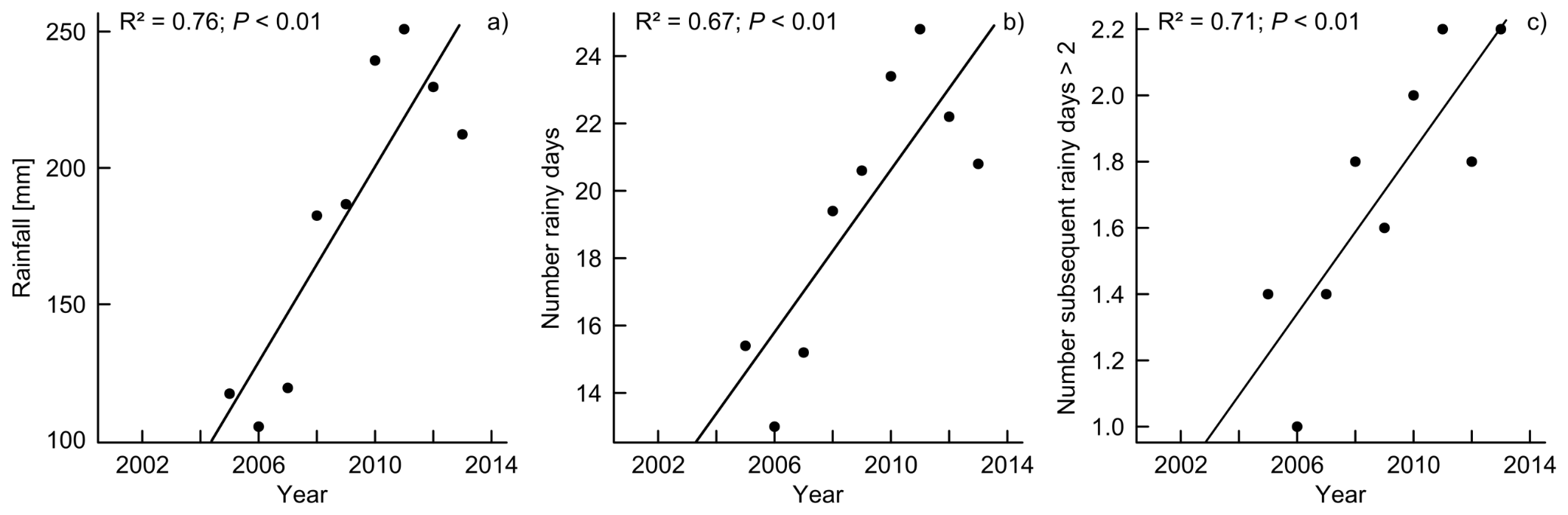


**Figure A1:** Rainfall on our study area between 2001 and 2014**.** a) five years moving average of annual rainfall, b) number of rainy days and c) number of periods with more than two humid days

**Figure A2:** Neighbour-effect intensity index (NInt_A_) based on the average tussock area of *Stipagrostis* in relation to the amount of seasonal rainfall (Spearman correlation coefficient r=0.31, p=0.54).

**Figure A3:** Soil moisture (%) in bare soil (solid line), active (dashed line) and inactive *Stipagrostis* tussocks (dotted line) within the first 10 days of a simulated rainfall event of 10 mm (n=15, three repeats). Bars indicate standard error.

**Table A1:** Habitat characteristics of sites unaffected and affected by *Crotalaria*. Differences in means tested by permutational t-tests, *t* and *p* values are given.

| Habitat characteristics | Unaffected | | | Affected | | | Unaffected vs. affected | |
| --- | --- | --- | --- | --- | --- | --- | --- | --- |
|  | Mean ± SE | Min | Max | Mean ± SE | Min | Max | *t* | *p* |
| Soil structure | | | | | | | | |
| Sand | 22.5 ± 1.3 | 15 | 30 | 24.0 ± 1.6 | 15 | 30 | 0.71 | 0.64 |
| Pebbles | 67.5 ± 1.5 | 60 | 75 | 65.5 ± 1.7 | 60 | 75 | -0.86 | 0.49 |
| Medium Stones | 2.2 ± 0.4 | 1 | 5 | 2.4 ± 0.3 | 1 | 5 | 0.37 | 1.00 |
| Large Stones | 1.6 ± 0.4 | 0 | 3 | 1.6 ±0.4 | 0 | 3 | 0.00 | 1.00 |
| Rock | 4.9 ± 0.6 | 3 | 10 | 4.6 ± 1.0 | 1 | 10 | -0.25 | 0.86 |
| Vegetation structure | | | | | | | | |
| Shrubs | 0.9 ± 0.4 | 0 | 3 | 1.0 ± 0.5 | 0 | 5 | 0.17 | 1.00 |
| Trees | 0.0 ± 0.0 | 0 | 0 | 0.0 ± 0.0 | 0 | 0 | - | - |
| *Crotalaria* 2009 | 3.0 ± 0.7 | 0 | 7 | 76.4 ± 12.3 | 24 | 166 | 5.96 | 0.001 |
| Grasses 2009 | 212.7 ± 14.3 | 139 | 268 | 210.1 ± 10.2 | 173 | 256 | -0.15 | 0.89 |

**Table A2:** *Crotalaria* seedling survival and number of *Crotalaria* measured during the interaction experiment.

| Day | *Crotalaria* | | *Crotalaria* and *Stipagrostis* inactive | | *Crotalaria* and *Stipagrostis* active | |
| --- | --- | --- | --- | --- | --- | --- |
| Day | *Crotalaria* survived | *Crotalaria* measured | *Crotalaria* survived | *Crotalaria* measured | *Crotalaria* survived | *Crotalaria* measured |
| 1 | 150 | 0 | 150 | 0 | 150 | 0 |
| 2 | 150 | 0 | 150 | 0 | 150 | 0 |
| 3 | 150 | 0 | 142 | 0 | 150 | 0 |
| 4 | 141 | 7 | 141 | 52 | 145 | 28 |
| 5 | 113 | 13 | 138 | 73 | 137 | 35 |
| 6 | 100 | 16 | 131 | 93 | 110 | 52 |
| 7 | 76 | 24 | 125 | 107 | 88 | 61 |
| 8 | 52 | 31 | 124 | 109 | 82 | 70 |
| 9 | 49 | 33 | 118 | 108 | 70 | 56 |
| 10 | 43 | 40 | 110 | 108 | 58 | 53 |
| 11 | 40 | 38 | 110 | 104 | 52 | 50 |
| 12 | 40 | 38 | 110 | 104 | 50 | 46 |
| 13 | 40 | 39 | 110 | 106 | 50 | 45 |
| 14 | 40 | 38 | 106 | 106 | 47 | 44 |
| 15 | 39 | 38 | 106 | 106 | 44 | 43 |
| 16 | 39 | 36 | 106 | 103 | 44 | 43 |
| 17 | 37 | 33 | 103 | 101 | 41 | 41 |
| 18 | 37 | 33 | 101 | 101 | 41 | 40 |
| 19 | 33 | 29 | 100 | 99 | 39 | 39 |
| 20 | 33 | 30 | 100 | 96 | 38 | 38 |
| 21 | 33 | 30 | 96 | 96 | 37 | 35 |
| 22 | 32 | 30 | 95 | 94 | 37 | 35 |
| 23 | 32 | 30 | 95 | 94 | 35 | 35 |
| 24 | 32 | 30 | 95 | 94 | 35 | 35 |
| 25 | 32 | 30 | 94 | 94 | 35 | 35 |
| 26 | 32 | 30 | 94 | 93 | 35 | 34 |
| 27 | 32 | 30 | 94 | 94 | 35 | 33 |
| 28 | 31 | 30 | 94 | 94 | 34 | 33 |
| 29 | 29 | 29 | 94 | 94 | 33 | 33 |
| 30 | 29 | 28 | 94 | 94 | 33 | 33 |
| 31 | 29 | 28 | 94 | 94 | 33 | 33 |
| 32 | 29 | 28 | 94 | 94 | 33 | 31 |
| 33 | 28 | 28 | 94 | 94 | 31 | 30 |
| 34 | 28 | 28 | 94 | 94 | 29 | 29 |

**Table A3:** Results of linear mixed effects models to analyse effects of day and treatment on *Crotalaria* height. Parameter estimates with standard error (SE), confidence intervals, *t* values with 1081 degrees of freedom, and *p* values are given. Treatment C: *Crotalaria*, Si: *Stipagrostis* inactive, Sa: *Stipagrostis* active, “:” indicates two-way interaction.

|  | Estimate ± SE | *t* | *p* |
| --- | --- | --- | --- |
| Day | 0.46 ± 0.01 | 53.09 | < 0.001 |
| Treatment C^a^ | -1.99 ± 0.36 | -5.47 | < 0.001 |
| Treatment C + Si^a^ | 1.79 ± 0.33 | 5.46 | < 0.001 |
| Treatment C + Sa^a^ | 1.47 ± 0.34 | 4.27 | < 0.001 |
| Day : Treatment C + Si^a^ | -0.06 ± 0.01 | -5.60 | < 0.001 |
| Day : Treatment C + Sa^a^ | -0.11 ± 0.01 | -9.56 | < 0.001 |

^a^ 0 was the reference level.

**Table A4:** Results of linear mixed effects models to analyze effects of day and treatment on *Stipagrostis* height. Parameter estimates with standard error (SE), confidence intervals, *t* values with 118 degrees of freedom for day 3 – 7 and 778 degrees of freedom for day 8 - 34, and *p* values are given. Treatment C: *Crotalaria*, Sa: *Stipagrostis* active, “:” indicates two-way interaction.

|  | Estimate ± SE | *t* | *p* |
| --- | --- | --- | --- |
| Day 3 - 7 |  |  |  |
| Intercept | 0.85 ± 0.75 | 1.13 | 0.26 |
| Day | 1.55 ± 0.11 | 14.70 | < 0.001 |
| Treatment Sa^a^ | -0.70 ± 1.07 | -0.64 | 0.53 |
| Day : Treatment Sa^a^ | 0.18 ± 0.37 | 0.15 | 0.23 |
| Day 8 - 34 |  |  |  |
| Intercept | 10.05 ± 0.83 | 12.08 | < 0.001 |
| Day | 0.16 ± 0.01 | 24.99 | < 0.001 |
| Treatment Sa^a^ | 1.14 ± 1.18 | 0.96 | 0.34 |
| Day : Treatment Sa^a^ | 0.12 ± 0.01 | 13.40 | < 0.001 |

^a^ Treatment C + Sa was the reference level.
